# Extracellular Vesicles in Facial Aesthetics: A Review

**DOI:** 10.3390/ijms23126742

**Published:** 2022-06-16

**Authors:** Li Ting Kee, Chiew Yong Ng, Maimonah Eissa Al-Masawa, Jhi Biau Foo, Chee Wun How, Min Hwei Ng, Jia Xian Law

**Affiliations:** 1 Centre for Tissue Engineering and Regenerative Medicine, Faculty of Medicine, Universiti Kebangsaan Malaysia, Jalan Yaacob Latif, Kuala Lumpur 56000, Malaysia; liting1027@hotmail.com (L.T.K.); chiewyongng@gmail.com (C.Y.N.); maimonah.almasawa@gmail.com (M.E.A.); angela@ppukm.ukm.edu.my (M.H.N.); 2 School of Pharmacy, Faculty of Health and Medical Sciences, Taylor’s University, Subang Jaya 47500, Malaysia; jhibiau.foo@taylors.edu.my; 3 Centre for Drug Discovery and Molecular Pharmacology (CDDMP), Faculty of Health and Medical Sciences, Taylor’s University, Subang Jaya 47500, Malaysia; 4 School of Pharmacy, Monash University Malaysia, Bandar Sunway 47500, Malaysia; how.cheewun@monash.edu

**Keywords:** extracellular vesicle, cosmetic, skin rejuvenation, anti-aging, anti-scarring, pigmentation

## Abstract

Facial aesthetics involve the application of non-invasive or minimally invasive techniques to improve facial appearance. Currently, extracellular vesicles (EVs) are attracting much interest as nanocarriers in facial aesthetics due to their lipid bilayer membrane, nanosized dimensions, biological origin, intercellular communication ability, and capability to modulate the molecular activities of recipient cells that play important roles in skin rejuvenation. Therefore, EVs have been suggested to have therapeutic potential in improving skin conditions, and these highlighted the potential to develop EV-based cosmetic products. This review summarizes EVs’ latest research, reporting applications in facial aesthetics, including scar removal, facial rejuvenation, anti-aging, and anti-pigmentation. This review also discussed the advanced delivery strategy of EVs, the therapeutic potential of plant EVs, and clinical studies using EVs to improve skin conditions. In summary, EV therapy reduces scarring, rejuvenates aging skin, and reduces pigmentation. These observations warrant the development of EV-based cosmetic products. However, more efforts are needed to establish a large-scale EV production platform that can consistently produce functional EVs and understand EVs’ underlying mechanism of action to improve their efficacy.

## 1. Introduction

Cosmetics have been used for thousands of years and have become a part of human lifestyles. They are applied to different parts of the human body to promote or modify the appearance to make it look more attractive. Among them, facial aesthetics is a form of cosmetic therapy that involves the application of non-invasive or minimally invasive techniques to improve facial appearance. Nowadays, consumers have a growing awareness about enhancing their facial appearance. They are paying more attention to the functionality and effectiveness over the price of the cosmetics [1]. Many efforts have been made to improve existing technologies and develop new technologies [2]. At the same time, cosmeceuticals, which are defined as cosmetic products that perform medical benefits for skin enhancement, are introduced [3].

Currently, nanotechnology is a rapidly growing field in cosmeceutical formulation design and development. This is because nanoparticles (NPs) can achieve otherwise unattainable goals using conventional technologies. NPs can improve the formulation by serving as a carrier for active ingredients. NPs have excellent skin penetration and tuneable release profile, thus enhancing the bioavailability and efficacy of the active ingredients, providing longer-lasting effects, and reducing the risk of adverse effects [2]. A combination of nanotechnology with cosmeceuticals that integrates active ingredients with skin improvement effects is known as nano-cosmeceuticals [4]. This approach allowed ready absorbance of active ingredients onto skin as they are packaged within the nanocarriers, subsequently exerting enhanced cosmetic and therapeutic effects to improve the skin appearance. The most commonly used nanocarriers in the cosmeceutical field include liposomes, niosomes, solid lipid nanoparticles, nanocapsules, micelles, dendrimers, and metal nanoparticles [5]. However, some of these NPs may produce toxicity or undesired side effects, such as the activation of the innate immune system, inflammation, and skin irritation, due to their composition, particle size, and charge [6].

In response to these issues, extracellular vesicles (EVs) are attracting much interest in the cosmeceutical field due to their suitable biological characteristics. EVs are lipid bilayer NPs released from almost all cell types into the extracellular space. Generally, EVs are classified into three types based on their size and biogenesis: exosomes (50–150 nm in diameter), microvesicles (MVs) (100–1000 nm in diameter), and apoptotic bodies (500–5000 nm in diameter) [7,8]. Exosomes are generated via the endosomal pathway, which involves inward budding of plasma membrane resulting in the formation of multivesicular bodies (MVBs). Then, a fusion of MVBs with the plasma membrane releases the intraluminal vesicles (ILVs) in MVBs into the extracellular space, giving rise to exosomes [9]. MVs are formed by local deformation and direct blebbing of the plasma membrane, while apoptotic bodies are formed due to cellular disassembly during programmed cell death [10,11].

**Figure 1:**
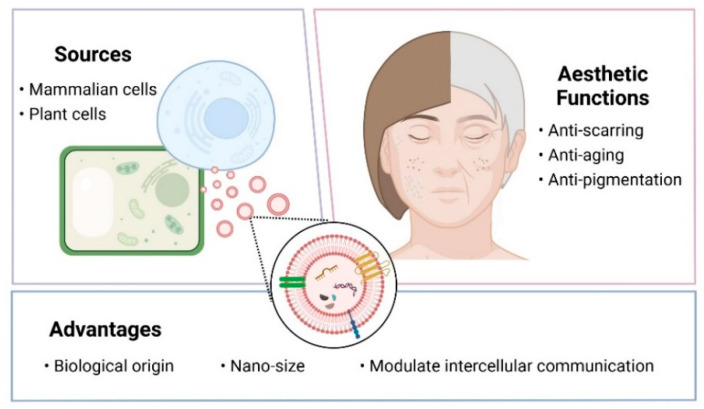
Overview of extracellular vesicles. Created with Biorender.com (accessed date: 12 May 2022).

EVs possess several features that make them ideal alternatives to other nanocarriers in cosmeceuticals to improve skin conditions. They naturally carry various biomolecules, such as proteins, lipids, DNA, and a repertoire of RNA species essential for intercellular communication and modulating recipient cells’ molecular activities [12,13]. Moreover, they are well tolerated in the body, easily taken up by cells, and can be targeted for uptake by specific tissues. EVs from both mammalian cells and plant sources have been therapeutically relevant in improving the skin condition, including the expedition of wound healing, reduction of skin pigmentation, wrinkles reduction, and scar formation prevention (Figure 1) [14,15,16,17]. These highlighted the potential of developing cosmeceutical products using EVs. In this review, we summarized the findings from the research that reported on the applications of EVs in facial aesthetics, i.e., as scar removal, rejuvenating the aging skin and reducing skin pigmentation. We also discussed the advanced delivery strategy of EVs, the therapeutic potential of plant EVs, and the findings of clinical studies using EVs to improve skin condition.

## 2. Applications of Extracellular Vesicles in Facial Aesthetics

**Figure 2:**
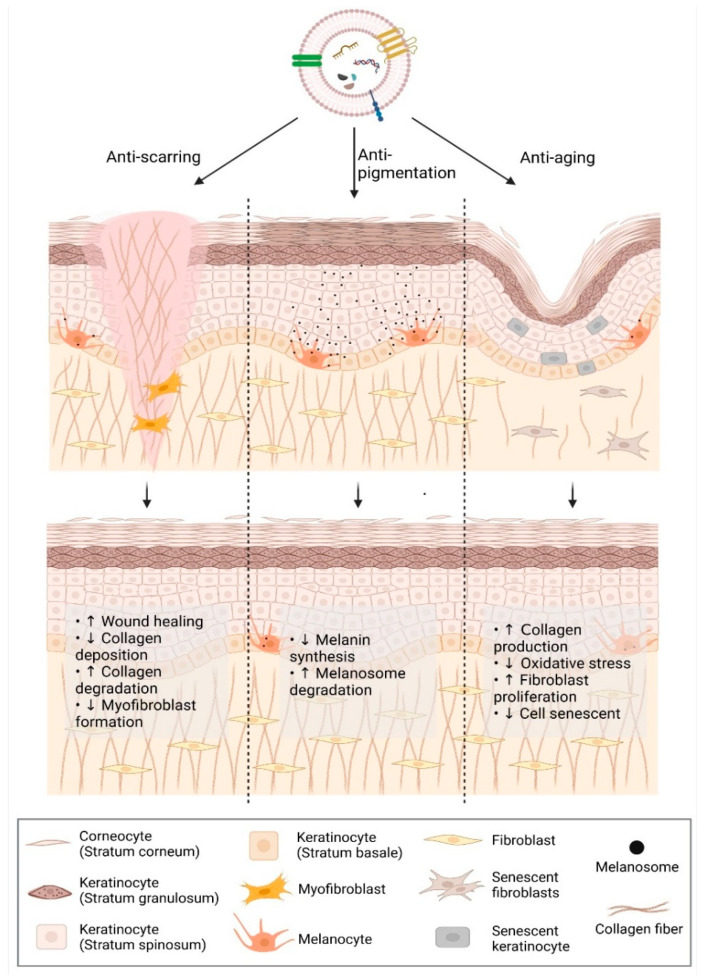
Extracellular vesicles in promoting anti-scarring, anti-aging, and anti-pigmentation effect. Created with Biorender.com (accessed date: 12 May 2022).

Anti-scarring, anti-aging, and anti-pigmentation are consumers’ most commonly sought functions in facial aesthetics. EV, a novel nanocarrier with various advantages, has been introduced to boost these effects in cosmeceuticals (Figure 2).

### 2.1. Anti-Scarring

#### 2.1.1. Scar Formation

Scars are associated with poor aesthetic appearance. Thus, mitigating scar formation is one of the main targets of cosmeceuticals. Scar formation is the outcome of three distinct but overlapping wound healing phases: the inflammatory, proliferation, and remodeling phases [18]. It involves interaction between multiple cell types, including keratinocytes, endothelial cells, fibroblasts, platelets and macrophages, and various biochemical factors produced by these cells [19]. Keratinocytes and fibroblasts are the most critical cell types that participate in wound healing by modulating the tissue regeneration processes, including extracellular matrix (ECM) deposition and remodeling [20].

Upon injury, the complement and coagulation cascades are activated to form a platelet plug to stop the bleeding. At the same time, the immune system is activated to initiate the inflammation, a hallmark of the inflammatory phase that lasts for two to three days. Then, the proliferative phase, which may last for three to six weeks, will occur. The proliferative phase is characterized by the development of granulation tissue and re-epithelialization. The proliferation of keratinocytes and migration of adjacent cells to the damaged tissues happen continuously until the wound is healed, concurrently with the formation of new blood vessels [19]. The new vessel formation is crucial, as it delivers oxygen and nutrition to meet the metabolic demand of the actively proliferating cells [21]. In the late proliferative phase, a portion of fibroblasts is activated and differentiated into myofibroblasts, producing abundant ECM. When the wound is closed, it enters the remodeling stage. At this point, excessive type III collagen (Col III) will be degraded and replaced with matured type I collagen (Col I). The remodeling phase will last from 2 weeks to more than 1 year [19]. The extent of scar formation is influenced by the intensity and duration of inflammation, the amount of collagen produced, and the deficiency in excessive collagen removal [22].

Fibronectin, decorin, and elastin are also involved in ECM remodeling. Fibronectin is essential in all stages of wound healing because it is involved in restoring tissue architecture and cellular processes such as adhesion, spreading, proliferation, migration, angiogenesis, and apoptosis. Moreover, fibronectin can create stable collagen I/III fibrillar network via an integrin-dependent mechanism, which is critical in restoring normal skin architecture [23]. The synthesis of elastin is critical during wound healing to ensure the reformation of elastic fiber network in the scar tissue to maintain the elasticity of the regenerated skin [24]. Decorin is a proteoglycan component that is involved in ECM construction and promotes cutaneous wound repair [25].

The inflammatory reaction is closely associated with scar formation. Macrophages are essential players in the transition from the inflammatory to the proliferative phases of wound healing. They modulate the wound healing process to control the extent of scar formation. They can stimulate ECM protein synthesis for tissue regeneration and healing and are closely associated with fibrosis [26]. In addition, several inflammatory chemokines and cytokines have been associated with fibrosis. Monocyte chemoattractant protein-1 (MCP-1) enhances wound healing by regulating the migration and infiltration of macrophages and monocytes [27] as well as stimulating collagen synthesis by fibroblasts [28] and the migration of endothelial cells [29]. Tumor necrosis factor-alpha (TNF-α), interleukin-1 beta (IL-1β), and interleukin-6 (IL-6) are pro-inflammatory cytokines produced by M1 macrophages that are linked to scar formation [30,31]. Tumor necrosis factor (TNF)-stimulated gene-6 (TSG-6) is released due to tissue damage and inflammation. It functions to suppress pathological scarring via inhibiting inflammation and reducing collagen deposition [32].

ECM is composed of many components, such as collagen, fibronectin, proteoglycans, laminin, elastin, hyaluronan, and glycoproteins. Collagen is the most abundant ECM of skin. Therefore, collagen synthesis and breakdown are the critical factors in scar formation. Transforming growth factor-beta (TGF-β), myofibroblasts, matrix metalloproteinases (MMPs), and tissue inhibitors of MMPs (TIMPs) are the primary regulators of collagen remodeling. Myofibroblasts are trans-differentiated from fibroblasts in response to skin injury and are characterized by the high expression of alpha-smooth muscle actin (α-SMA). Myofibroblasts play a crucial role in cutaneous wound healing by stimulating collagen synthesis, notably Col I and Col III for ECM remodeling [33]. The persistent presence of myofibroblasts during the later phases of wound healing is known to cause scar formation [34,35]. Myofibroblasts are mainly regulated by the TGF-β isoforms, particularly TGF-β3 and TGF-β1. TGF-β3 acts as a scar prevention and reduction factor due to its ability to inhibit myofibroblast differentiation, whereas TGF-β1 promotes myofibroblast differentiation and granulation tissue formation that lead to scar formation [36].

TGF-β is also known to regulate the expression of MMPs. MMPs are a group of zinc-dependent extracellular proteinases which remodel the ECM. In scar tissue, lower expression of MMPs results in collagen deposition, which leads to scar formation [37]. There are three predominant groups of MMPs: collagenases, gelatinases, and stromelysins, responsible for degrading basement membrane collagen and denatured structural collagens [38]. MMP-1 and MMP-3 can be used as scar formation indicators. MMP-1 facilitates keratinocyte migration on collagen, which is vital in initiating the re-epithelialization process [39]. In addition, MMP-1 also breaks down the excessive collagen matrix to mediate collagen remodeling during wound healing and wound bed maturation [40]. MMP-3, also known as stromelysin-1, involves the degradation of basement membrane collagen and activation of MMP-1. In addition, MMP-3 exerts a unique function in the early healing phase by initiating wound contraction, which involves interaction between wound fibroblasts and the surrounding ECM [41]. Generally, MMPs inhibit collagen deposition during ECM remodeling and contribute to scarring resolution [40]. However, the biological activity of MMPs can be countered by TIMPs, especially TIMP-1 and TIMP-2 [42]. The ratio of MMPs and TIMPs has been suggested to promote scarless restoration [43].

#### 2.1.2. Extracellular Vesicles in Reducing Scar Formation: Evidence and Clues

Several options, including surgery, laser therapy, chemical peels, dermabrasion, injection with steroid or collagen, and ointment, are available to minimize or remove scarring. EVs have gained interest as a biologic to reduce and remove scarring, regulate inflammation, and stimulate cell migration, proliferation, and matrix reconstruction. Many studies have been performed to investigate the role of EVs in scar reduction (Table 1). Overall, most studies revealed that EVs could accelerate wound healing and promote scarless healing with well-reorganized collagen fibers with less cross-linking and a more flattened epidermis surface. The studies also showed that EVs could promote skin cells’ proliferation, migration, and angiogenesis.

Li et al. demonstrated that adipose tissue-derived mesenchymal stem cell (ADSC)-derived EVs inhibited the migration and proliferation of hypertrophic scar tissue-derived fibroblasts (HSFs) in vitro and reduced the collagen deposition in a mouse full-thickness wound model to attenuate fibrosis [33]. These results contradict studies that found that MSC-derived EVs could increase collagen deposition and stimulate fibroblast proliferation and migration during wound healing [14], indicating that EVs exerted different influences on cells in normal and pathological conditions.

As stated before, the regulation of collagen synthesis is the determining point in scar formation. However, the collagen expression patterns varied between the studies. While some studies found that EV treatment could help to increase Col I and Col III synthesis [45,46], others have shown increased Col III and decreased Col I production [36]. On the other hand, some studies revealed that EV treatment decreased Col I synthesis [44,53,65], and some found that both Col I and Col III synthesis were reduced with EV therapy [32,33,49,50]. Several factors might influence the discrepant results. Firstly, collagen synthesis depends on the stages of wound healing. In the early stage of healing, collagen synthesis is necessary to form granulation tissue that supports skin regeneration. While in the remodeling phase, collagen deposition is reduced to avoid excessive scar tissue formation. Moreover, the ratio of Col I: Col III is also an important criterion during ECM remodeling to restore normal skin architecture [17,36]. In the initial stage of healing, the ratio of Col III to Col I expression increases, and the ratio will decrease during scar maturation [23]. Secondly, variations in EV source, preparation and dosage used in every study also contributed to the discrepant findings.

Collagen synthesis is chiefly modulated by myofibroblasts, TGF-β3, TGF-β1, MMPs and TIMPs. Many studies showed that α-SMA expression was reduced after EV treatment, indicating the reduced myofibroblast differentiation. Besides, EV treatment decreased the TGF-β1 level [20,32,36,44,51] and increased the TGF-β3 level [36,52]. Furthermore, some studies showed that EV treatment increased the expression of MMP-1 [44,46,50] and MMP-3 [36,47]. A study found that MMP-2 and MMP-14 were downregulated and MMP-13 increased with EV treatment during fibroblast–myofibroblast transition [54]. MMP-2 and MMP-14 involve in collagen remodeling, whereby they degrade the collagen and elastin fibers [38]. MMP-13 is an interstitial collagenase that coordinates cellular activities such as the motility and contractility force important in the growth and maturation of granulation tissue [66]. TIMP, as the regulator of MMPs, also has been investigated. Zhao et al. found that the expression of TIMPs was slightly increased, while Zhang et al. and Wang et al. reported a reduction in TIMPs with EV treatment. Nonetheless, the ratio of MMP to TIMP was found to increase in these studies. These results are consistent with other studies that showed that a higher MMP: TIMP ratio could promote scarless repair [37,43]. EV treatment also leads to an increase in the expression of elastin [46,53], as well as the expression of fibronectin [46,54] and decorin [54] in fibroblasts.

Intense inflammation has been linked with excessive scar tissue formation [67]. Jiang et al. reported that exosomes derived from TSG-6 modified MSCs suppressed scar formation during wound healing by subsiding the inflammation, as indicated by the reduced levels of MCP-1, TNF-α, IL-1b, and IL-6 in the scar tissues [32]. However, Tutuianu et al. showed that exosomes derived from bone marrow-derived MSCs (BM-MSC-Exos) insignificantly reduce the inflammatory cytokine expression, i.e., TNF-α, of the activated macrophages. Therefore, further investigation is needed to investigate the role of EVs in cutaneous wound inflammation.

In summary, these data provide insights into the regenerative properties of EVs in promoting scarless healing. It is believed that EV could be applied in all phases of wound healing to reduce scar formation.

### 2.2. Anti-Aging

#### 2.2.1. Skin Aging

Skin aging is a natural process reflected through changes in appearance such as wrinkles, sagging, fragility, and impairment in skin tone and texture. There are two types of skin aging, which are intrinsic aging (age-dependent aging) and extrinsic aging (environmental factor-mediated aging) [68]. Both aging types showed weakened dermal structure with poor mechanical integrity. This is because dermal fibroblasts gradually lose their regenerative capacity and ability to synthesize structural components such as collagen, elastin, and fibronectin [69]. Moreover, aging skin has poorer epidermal renewal capacity, which results in epidermal thinning. Ultraviolet (UV)-induced DNA damage in keratinocytes resulted in aberrant cell proliferation [15,55]. In addition, the photoaged skin also showed the characteristics of uneven epidermal thickness and pigmentation, capillary regression and disorganization, decreased collagen matrix, accumulation of reticular fibers, elastic fibers denaturation, and sebaceous gland hyperplasia [55].

The balance between collagen synthesis and breakdown is critical to maintaining skin renewal and youthfulness. Col I and Col III account for 80–85% and 10–15%, respectively, of the total collagen in the skin [70]. With age, the proportion of Col I declines whilst the amount of Col III increases, especially in ultraviolet B-rays (UVB)-exposed areas [55]. Besides, the MMPs level will increase, and the TIMPs level will decrease in extrinsic and intrinsic aging [71]. MMPs are activated by the reactive oxygen species (ROS) to fragment and degrade the ECM proteins [72]. TIMPs involves in skin aging indirectly by regulating the MMPs. In addition, TIMPs also have been shown to suppress apoptosis and enhance the proliferation of fibroblasts [73,74]. The changes in MMPs and TIMPs levels with age lead to impaired collagen homeostasis. Besides, the expression of TGF-β that is involved in cell proliferation, differentiation, migration, and matrix synthesis is reduced in the photoaging skin due to the interference in the TGF-β signaling pathway [75]. All these changes lead to collagen and elastic fibers disorganization. Eventually, the skin becomes slack and collapses, resulting in wrinkle formation.

Besides that, inflammatory and pro-apoptotic factors are also involved in skin aging. They are elevated in senescence fibroblasts, resulting in the production of MMPs that drive ECM destruction [76,77,78]. Furthermore, the inflammation and ROS act synergistically to promote MMP activation, exacerbating the ECM damage [15]. Apart from the degradation of ECM protein, ROS also causes oxidative denaturation of macromolecular substances, such as nucleic acids and proteins, leading to DNA damage, cell cycle arrest, and cell apoptosis [61].

#### 2.2.2. EVs in Anti-Aging: Evidence and Clues

In the past, several anti-aging strategies, i.e., cosmetics, chemical peel, phototherapy, and micro-needling, have been introduced to improve the appearance of aging skin [79]. However, the improvement observed does not last for long. As a result, EVs have been investigated as a potential candidate that might provide better results, as they play a substantial role in influencing many processes involved in skin aging (Table 1).

EVs have been reported to promote the migration and proliferation of fibroblasts in many studies [15,59,61,80]. Deng et al. and Xu et al. found that EV therapies rescued the fibroblasts from cell cycle arrest and decreased the expression of cell cycle arrest-related proteins P53 and P21 [15,58]. Oh et al. showed that human iPSC-derived exosomes (iPSC-Exo) abrogated the production of senescence-associated β-galactosidase (SA-β-Gal), which is a biomarker of senescent fibroblasts [59]. Similarly, Deng et al. observed that EV therapy protected the fibroblasts from UVB-induced senescence [58]. These findings showed that EV therapy could prevent fibroblast senescence and restore the regenerative capability of aging fibroblasts. Adipose-derived stem cell extracellular vesicles (ADSC-EVs) promote epidermal cell proliferation, decrease the epidermal thickness and increase dermal thickness [15]. On top of that, Liang et al. demonstrated that ADSC-EVs also corrected the abnormal thickening of the epidermis and aberrant proliferation of stratum basale cells [55].

Various studies also showed the elevation of Col I expression and the reduction of MMPs expressions with EV treatment [15,55,58,59,60,80]. However, contradictory findings have been reported for Col III expression, whereby Choi et al. and Go et al. reported an increment in the expression of this protein whilst Liang et al. saw a reduction. This discrepancy might be due to the variation in the models used (in vitro model vs. in vivo model). Therefore, further research using the in vivo model is required to confirm changes in Col III expression. Choi et al. extended that EV treatment can intervene in the UVB-mediated decrease in Col II and V [80]. In addition, the decreased elastin and fibronectin [60,80], as well as TGF-βs and TIMPs [80] induced by UV irradiation, were also restored by EV treatment.

Besides, EV treatment reduces ROS generation that mediated UV-induced skin aging [15,58,59,62]. Wu et al. found that treatment with umbilical cord-derived MSC exosome (UC-MSC-Exo) healed the UV-radiation induced skin photodamage in vivo by speeding up the ROS clearance, promoting autophagy activation, improving DNA repairability, and reducing cell apoptosis [61]. The authors attributed the improvement to the 14-3-3ζ protein delivered by the UC-MSC-Exo, which enhanced the expression of keratinocytes’ Sirtuin 1 (SIRT1) under oxidative stress conditions. In another study, Deng et al. discovered that EV treatment elevated the expression of glutathione peroxidase 1 (GPX-1) to reduce oxidative stress and UVB-induced cell aging [58]. On the other hand, Xu et al. discovered that ADSC-EV treatment reduced ROS generation by boosting the expression of the antioxidant enzymes, i.e., superoxide dismutase type 1 (SOD-1) and catalase (CAT) [15]. In addition, ADSC-EV treatment also attenuated macrophage infiltration stimulated by UVB and suppressed M0 to M1 macrophage polarization in response to activation by proinflammatory stimuli. Besides, ADSC-EVs also suppressed the activation of the Nuclear factor kappa B (NF-κB) 1 signaling pathway that regulates inflammatory cell proliferation, activation, and cytokine production. These results showed that EV treatment could upregulate the expression of antioxidant enzymes to reduce the oxidative stress induced by UVB irradiation.

In short, EV treatment exerted an anti-aging effect by enhancing fibroblast activities (higher proliferation and migration), regulating collagen turnover (higher TIMPs and lower MMPs), attenuating ROS production (higher concentration of antioxidant enzymes), and reducing tissue inflammation.

### 2.3. Anti-Pigmentation

#### 2.3.1. Skin Pigmentation

Hyperpigmentation is a common skin problem that affects people of all skin types. Generally, skin pigmentation happens through the synthesis and distribution of melanin particles between melanocytes and keratinocytes in the epidermal layer [63]. Melanin is synthesized to defend the human skin from damaging UV radiation, toxic chemicals, and other environmental variables and in response to intracellular factors such as keratinocytes-derived cytokines [64]. However, excessive melanin production causes hyperpigmentation and cutaneous issues such as freckles, age spots, and melasma. There are three essential processes involved in skin pigmentation: melanin synthesis, melanosome transportation to adjacent keratinocytes, and melanosome degradation [63].

Melanin production is controlled by numerous intracellular signaling systems, with cyclic adenosine monophosphate (cAMP)-dependent signaling pathways serving as the primary driver of melanogenesis. When keratinocytes are exposed to UV, they will release alpha-melanocyte-stimulating hormone (α-MSH), which will bind to the melanocortin 1 receptor (MC1R) on the melanocyte surface, resulting in activation of cAMP cascades. Activation of cAMP will then induce the expression of microphthalmia-associated transcription factor (MITF). MITF is a master regulator for melanogenesis-related proteins, including tyrosinase (TYR), tyrosinase-related protein 1 (TYRP1), and tyrosinase-related protein 2 (TYRP2), which are critical in melanin synthesis [81]. TYR is a multifunctional copper-dependent enzyme that catalyzes the conversion of L-tyrosine to Levodopa (L-DOPA), which is the rate-limiting step in melanin production [82]. TYRP1 activates and stabilizes TYR for melanin synthesis [83], whereas TYRP2 is involved in the early phases of melanin synthesis and melanocyte development, survival, and function [84]. They are found in the membrane of melanosomes and interact with one another to regulate melanin formation [85]. Keratinocyte is the final destination of melanosomes. Thus, the total number of melanosomes in keratinocytes significantly impacts skin pigmentation. Autophagy is an essential regulator of skin pigmentation by regulating the melanosome degradation in keratinocytes and melanocytes [86].

#### 2.3.2. Extracellular Vesicles in Regulation of Skin Pigmentation: Evidence and Clues

Recently, evidence has demonstrated that EV treatment can slow down UVB-stimulated melanin production and promote melanosome degradation (Table 1). This showed that EVs contain factors that can avoid skin hyperpigmentation. Keratinocyte- and amniotic stem cell-derived EVs have been found to reduce cellular melanin content by lowering the critical melanogenesis-related proteins (TYR, TYRP1, and TYRP2) and MITF that regulates these proteins [62,63]. In addition, Wang et al. also discovered that EVs promote melanosome degradation by activating autophagy in the Murine melanoma cell line from a C57BL/6J mouse (B16F10 melanoma cells) [63]. The authors attributed the anti-pigmentation effects of EVs to the miR-330-5p, miR-181a-5p, and miR-199a content. Besides, UV exposure induced EV production by melanocytes [64]. The melanocyte-derived EVs induced the anti-apoptotic signaling and enhanced the proliferation and migration of keratinocytes [64]. These results revealed that EVs could reduce skin pigmentation under normal conditions and act as paracrine factors to lessen the damage brought by UV exposure.

## 3. Advanced Delivery Strategy for EVs

**Figure 3:**
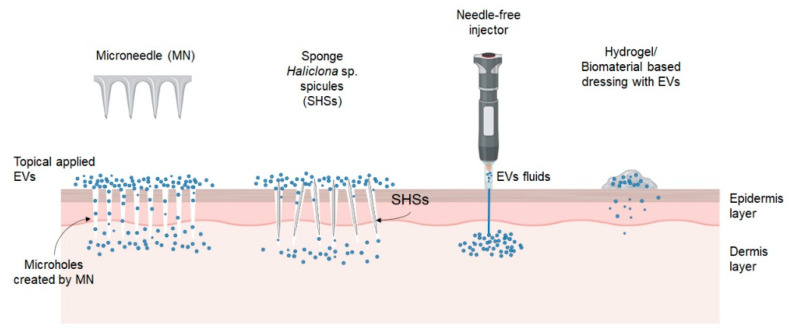
Advance delivery strategy for EVs. Created with Biorender.com (accessed date: 12 May 2022).

Although EVs are absorbed by human skin when applied topically, their penetration is limited to the stratum corneum, whereby less than 1% of the EVs were found to penetrate the stratum corneum and localized in the stratum granulosum [87]. Improving skin penetration is critical to maximizing the function of EVs applied topically. Therefore, various physical and chemical penetration enhancing technologies were explored to increase the absorption of EVs into the skin, including microneedles, and incorporated with hydrogel and scaffold (Figure 3).

### 3.1. Physical Penetration

Microneedles (MN) or micro-needling device consists of multiple micron-sized needles has been used for transdermal delivery of drugs with the advantages of being safe, painless, convenient, and non-invasive (Table 2). It allows drugs to directly reach the dermal tissue by creating plenty of microholes to bypass the stratum corneum barrier [88]. Cao et al. found that a combination of microneedle and ADSC-EVs exhibited promising anti-aging effects in photoaging skin in vivo, as indicated by the least wrinkles, the maximum collagen density, and the most organized collagen fibers compared to the other groups [89]. There is also a decrease in epidermal thickness and improved skin barrier function with higher stratum corneum hydration values and lower trans-epidermal water loss (TEWL).

In another study, marine sponge *Haliclona* sp. spicules (SHSs), a novel kind of microneedles in nature, were used to enhance skin delivery of hydrophilic biomacromolecules. Using SHSs, over 1000 microchannels could be created per mm^2^ of skin through simple massage [91]. These microchannels allow UC-MSC-Exo to penetrate into the skin in vivo to repair the photoaging skin by decreasing epidermal hyperplasia and restoring the wavy dermal-epidermal junction [90]. In addition, combined treatment with SHSs and UC-MSC-Exo reduced the micro wrinkles and gene expression of MMP-1, and increased the gene expression of Col I, elastin, and fibronectin. The authors attributed the improvements to SHSs, which considerably increase skin absorption of exosomes, whereby most of the exosomes were found in the deeper skin layer.

Alternatively, a needle-free jet injector also can be used to deliver drugs across the skin, through the epidermis and deep into the dermal layer using a high-pressure jet of fluid medication [92]. This new injection method is superior to the conventional syringe-based technique, whereby it can reduce pain and harm and provide greater penetration and absorption. The needle-free jet injector successfully increased the entry of exosomes into the dermis in a manner that is more effective than topical application [69]. In vivo, the UVB-induced skin photoaging began to show visible results three weeks after receiving a single EV injection. The EV-treated skin has the most superficial and thinner wrinkles, plentiful collagen fibers, compact stratum corneum, and thinnest epidermal layer.

These findings suggested that MN, SHSs, and needle-free injectors create pores that allow EVs to penetrate through the epidermis and reach the dermis to enhance the therapeutic efficacy of EVs in reversing photoaging.

### 3.2. Hydrogel/Biomaterials-Based Dressings

Apart from limited skin penetration, EVs have a relatively short half-life in skin because of their rapid clearance by body fluids such as sweat and exposure to external stimuli. As a result, establishing a sustained delivery mechanism for EVs is critical to optimizing the therapeutic effect of EVs in the skin. Hydrogels have been frequently used for the sustained release of pharmaceuticals. They are hydrophilic polymers that can swell in water and hold a high amount of water while keeping their structure. Hydrogels are highly porous, can hold a high concentration of pharmaceutical agents, and can be used to facilitate controlled release by fine-tuning their physicochemical properties [93]. Recent research has investigated the potential of using natural and synthetic hydrogels such as alginate (Alg), chitosan (CS), fibrin, gelatin, poloxamer 407, and polyethylene glycol (PEG) to deliver EVs to the skin (Table 3). Generally, better skin improvement was seen when hydrogels were used as EV carriers.

Alg is a polysaccharide polymer found in marine brown algae and has been widely used for the controlled release of drugs. Its advantages include having excellent biocompatibility, being easy to prepare, low cost, and pharmacologically inert (biocompatible and not influencing the pharmacological effect of drugs), and being able to be administered via minimally invasive techniques to achieve the continuous release of encapsulated drugs [100]. Due to these advantages, a few recent studies used Alg-based hydrogels for the cutaneous delivery of EVs. Shafei et al. used Alg hydrogels to preserve the exosomes at the wound site in a full-thickness excisional wound model in rats. The hydrogel permitted sustained release of ADSC-Exo for up to 172 h. Consequently, significant improvements in wound closure, collagen synthesis, and vessel formation were recorded in wounds treated with Alg-Exo hydrogel [94]. In other studies, Alg hydrogel was combined with synthetic polymers to enhance its physicochemical properties to prevent sudden burst release that causes drug leakage and overdose [101]. Shen et al. prepared bilayered thiolated Alg/PEG diacrylate (BSSPD) hydrogel. They used it to sequentially deliver BMSC-EVs (normal BMSC-EVs at the upper layer and miR-29b-3p-enriched EVs at the lower layer) to promote full-thickness wound healing in rats and rabbit ears [17]. The result showed that the EV-loaded BSSPD hydrogel was compatible and could promote wound healing. The healed wounds showed more systematic collagen organization (higher thickness with reduced Col I/III ratio) and vasculature with less fibrosis (lower protrusion height and scar elevation index).

CS hydrogels are also used to achieve the sustained release of active ingredients. CS is a natural poly-cationic biopolymer that is biocompatible and biodegradable [102]. In addition, it also possesses features such as thermal sensors, loose porous microstructure antimicrobial, mucous adhesive, and non-toxic that render it suitable for topical drug delivery [103]. The authors found that the delivery of exosomes derived from human endometrial stem cells (hEnSCs-Exo) incorporated in CS-glycerol hydrogels significantly reduced the healing time and enhanced re-epithelialization, improved formation of skin appendages, reduced immature granulation tissue, and promoted vascularization and angiogenesis [95]. Another study also revealed that CS hydrogels could extend the release of EVs and significantly improved EV retention in vivo [68]. After CS-EV treatment, the aging skin demonstrated signs of rejuvenation, whereby the authors observed higher collagen expression and tissue structure restoration. In addition, CS-EVs exerted an anti-aging effect by promoting ECM remodeling whereby the expressions of Col I, Col III, TIMP-1, and TIMP-2 increased, and the expressions of SA-β-gal, MMP-1, MMP-2, MMP-3, and MMP-9 decreased. Carboxymethyl CS (CMCS) is a CS derivative that shares similar properties as its parent compound but with water solubility in a wider pH range. In the study of Li et al., UC-MSC-Exo was loaded in CMCS/poloxamer 407 hydrogels crosslinked with genipin [96]. The hydrogels showed sustained release of UC-MSC-Exo. In vivo wound healing study showed that the EV-loaded CMCS/poloxamer 407 hydrogels accelerated wound healing, promoted regeneration of dermal appendages, stimulated collagen deposition and organization, and subsided inflammation by attenuating TNF-α and IL-1β expressions.

Gelatin methacryloyl (GelMA) hydrogel is a gelatin derivative widely used in various biomedical applications due to its ability to mimic the ECM properties and customizable physical characteristics [104]. Zhao et al. combined GelMA hydrogel with human umbilical vein endothelial cells-derived exosome (HUVEC-Exo) and applied it to the full-thickness cutaneous wounds. They found that the hydrogel could encapsulate and promote the sustained release of HUVEC-Exo. The treated wounds had faster re-epithelialization, improvement in collagen maturity, and better angiogenesis [97].

Fibrin gel (FG) is a biodegradable biomaterial prepared from fibrinogen and thrombin and is often used to stop bleeding and promote wound healing. FG can be locally injected, permit controlled release of active ingredients, and enhance wound healing. Thus, it is an ideal drug carrier. Oh et al. incorporated L929 murine fibroblast cell line-derived EVs (L929-EVs) in FG to facilitate retention of L929-EVs at the wound site. The results showed that L929-EVs loaded FG supported scarless wound healing in a full-thickness wound model via the acceleration of the wound closure rate, the promotion of collagen formation and maturation, and the simulation of angiogenesis [98].

HydroMatrix is a synthetic peptide nanofiber scaffold developed for cell culture and tissue engineering. It can self-assemble into a three-dimensional hydrogel in response to changes in temperature and ionic strength. HydroMatrix can sustain the proliferation of many cells and has been used as a carrier to retain EVs on the target site [105]. According to Duan et al., HydroMatrix loaded with epidermal stem cells-derived exosome (EPSC-Exo) hastened wound healing and reduced scar formation in a full-thickness wound model [99]. Besides, EPSC-Exo also promoted the regeneration of skin appendages, nerves, and vessels, stimulated the normal distribution of collagen, and reduced myofiber formation by inhibiting the TGF-β1 expression. In a separate study, HydroMatrix carrying three-dimensional (3D) cultured perivascular cell-derived EVs (PVC-EVs) significantly improved wound contraction, activation of myofibroblasts, and collagen deposition of full-thickness skin defect model in rats compared to HydroMatrix with two-dimensional (2D) cultured PVC-EVs [12]. In addition, PVC-EVs also enhanced the expression of vascular endothelial growth factor (VEGF) and angiogenesis.

In short, the combination of EV with hydrogels or biomaterial-based scaffolds has promoted scarless wound healing and anti-aging effects, which are essential in the cosmetic field for aesthetic purposes. Although many hydrogels or biomaterial-based scaffolds have been utilized in animal experiments to facilitate controlled delivery of EVs and EV retention at the target region, the EV/scaffold combination is still not commonly used in clinical practice. Therefore, more clinical studies should be carried out to prove the safety and efficacy of EV/scaffold combination in skin aesthetic application.

## 4. Plant EVs in Skin Improvement

Plant EVs were discovered in the 1960s, but little is known about them. A recent review suggested that plant EVs are involved in plant immune system modulation, plant defense response, plant-microbe symbiosis, and mediate intercellular communication and cross-kingdom communication by shuttling RNAs, proteins, and bioactive compounds [106,107]. Plant EVs share many properties with mammalian and bacterial EVs, and they have huge therapeutic potential, including anti-inflammatory, anti-tumor, mammalian microbiota modulation, and drug delivery [108]. Studies explored the application of plant EVs in skin diseases and found that they promoted skin regeneration just like mammalian EVs [109]. These findings aroused increasing interest in plant EVs in the cosmeceutical field, highlighting them as an ‘animal-free ingredient’ [107]. Research on various sources of plant-derived EVs was summarized in Table 4.

Ingredients from marine sources have been discovered to have various biological activities, including the regulation of skin pigmentation. EVs from *Codium fragile* and *Sargassum fusiforme* have been shown to reduce α-MSH-mediated spots and downregulate melanogenesis-related proteins, namely TYR, TYRP-1, and MITF in human melanoma cell line (MNT-1) [16]. Both EVs were found to exert anti-melanogenic effects by inhibiting melanogenesis in the epidermal basal layer in a 3D epidermal model. A clinical study conducted on 21 healthy female subjects found that applying a prototype cream with 5 µg/mL of *C. fragile* once a day for 4 weeks improved skin whitening by 0.94% and 1.31% at two weeks and four weeks, respectively.

In a different study, researchers investigated the anti-melanogenic effects of EVs derived from the leaves (LEVs) and stems (SEVs) of *Dendropanax morbifera* and found that both EVs exhibited no cytotoxicity and exerted a whitening effect in the Mouse cell line derived from melanoma (B16BL6 melanoma cell) by reducing the melanin levels [85]. However, LEVs demonstrated better anti-melanogenic effects compared to SEVs. LEVs regulated genes and proteins related to melanogenesis and significantly inhibited the melanin synthesis in a human epidermis model.

Aloe vera is one of the most extensively used antioxidant sources in the cosmetology and medical field due to its anti-inflammatory, anti-aging, antibacterial, antitumor, and pro-healing effect [111]. A recent study identified that the peel of Aloe vera exhibited more antioxidant activity than other sections [112]. Thus, EVs from Aloe vera peels (A-EVs) were isolated and investigated [107]. A-EVs showed good cytocompatibility on human skin cells, reduced the level of intracellular reactive oxygen species (ROS), and significantly upregulated the antioxidant defense signals, i.e., nuclear factor erythroid 2-related factor 2 (Nrf2), heme oxygenase-1 (HO-1), CAT, and SOD, of keratinocytes. Moreover, A-EVs enhanced the migration of keratinocytes and fibroblasts. These findings reveal that A-EVs could promote skin rejuvenation and regeneration by activating the antioxidant defense mechanisms and promoting wound healing.

*Panax ginseng* is a traditional herbal medication used primarily in East Asian countries. Their identified benefits included antimicrobial, anti-inflammatory, anti-cardiovascular disease, anticancer and neuroprotective, and others because of its ginsenoside content [113]. The most well-studied components of *P. ginseng* are ginsenosides; however, it also contains other constituents that were thought to have pharmacological effects [114]. In a study, EVs from ginseng roots (GrEVs) and the culture supernatants of ginseng cells (GcEVs) derived from *P. ginseng* were isolated and tested on human skin cells [110]. Both EVs demonstrated anti-senescence and anti-pigmentation effects and had no cytotoxicity on UVB radiated human skin cells by downregulating SA-β-Gal activity, senescence associated markers, and melanogenesis-related proteins.

To sum up, plant EVs displayed cytocompatibility, anti-aging, whitening, and pro-healing properties suitable for cosmeceutical applications. However, the current evidence is limited to in vitro 2D and 3D skin model experiments. The results need to be validated through clinical studies.

## 5. Clinical Study of EVs

To date, very few clinical studies have been performed to determine the potential of EVs as cosmeceuticals, even though encouraging findings have been reported in many in vitro and preclinical studies. On clinicaltrials.gov, there are no clinical studies on EVs in skin improvements such as anti-scarring, anti-aging, and anti-pigmentation.

In the clinical study by Cho et al. (Table 5), the topical application of a ADSC-Exo formulation demonstrated a skin brightening effect on volunteers with hyperpigmentation [115]. The melanin level was reduced four weeks after treatment, and no adverse effects were observed. In another clinical study by Kwon et al., ADSC-Exo applied on atrophic acne scars treated with a fractional carbon dioxide laser (FCL) reduced the FCL’s adverse effects by accelerating tissue regeneration and wound healing [116]. ADSC-Exo treated sides showed greater improvement with lower échelle d’évaluation clinique des cicatrices d’acné (ECCA) scores and Investigator’s Global Assessment (IGA) scores compared to the control sides. In addition, the ADSC-Exo side also showed a significant reduction in the number of atrophic scars, mean pore volume, and skin surface roughness. ADSC-Exo treatment reduced the severity of adverse effects, i.e., erythema, pain, edema, and dryness, which resolved in 5 days. These results suggested that ADSC-Exo can not only be used as an adjuvant to improve the therapeutic efficacy of existing therapies but also shortens the recovery time and reduces the adverse effects.

## 6. Limitation and Prospective

Altogether, numerous evidence showed that EVs possess biological activities that render them suitable and effective for aesthetic applications. However, there are significant barriers to their commercialization. The main roadblock is developing scalable production and storage methods for EVs. Naturally, the quantity of EVs secreted by the cultured cells is very low. Thus, techniques such as 3D culture using a bioreactor, physical stimulation (e.g., radiation and mechanical), chemical stimulation (e.g., drugs and small molecules), genetic manipulation (e.g., modulation of EV biogenesis and release pathway), and physiological modification (e.g., hypoxia and nutrient deprivation) are being explored to upscale the EV production [117]. Additionally, EV-mimetic nanovesicles can be produced on large-scale using techniques such as nitrogen cavitation [118], sonication [119], and porous membrane extrusion [120]. However, the cargoes of the produced EVs are influenced by the culture environment and protocol. Thus, it is essential to characterize the EVs’ cargo and examine its therapeutic effects.

Multiple isolation methods can isolate the EVs secreted by the cells. Generally, to various extents, all these techniques have the issues of contamination, time and cost consumption, and instability [121]. Therefore, a rapid and straightforward isolation approach capable of producing a high number of pure EVs should be secured. Storage of EV-based cosmeceuticals is another hurdle, as EVs are not stable at room temperature, 4 °C, and −20 °C [122]. EVs are more stable at −80 °C. However, this storage condition is unfavorable, as it increases the cost of storage and transport. Thus, innovations in packaging or formulation are needed to increase the shelf-life of EV-based cosmeceuticals at room temperature. Lyophilization showed potential in enhancing EV stability [123].

The next difficulty is the inability to achieve an appropriate topical application concentration of EVs for enhancing skin rejuvenation and regeneration. Many studies have employed different concentrations of EVs in skin models. However, there is no standardized concentration for skin rejuvenation and treating skin diseases. Although it has been demonstrated that EVs are safe for topical application and do not cause inflammation and skin sensitization [124], the optimal dose still needs to be determined to ensure that EVs are safe to be used regularly and there are no long-term side effects from topical administration.

EVs need to overcome the skin barrier and reach the deeper skin tissues to exert their biological functions. However, EVs have poor penetration through the stratum corneum when applied topically. Physical penetration has been proven to be an effective way to bypass the epidermal barrier in many transdermal drug delivery experiments. Nevertheless, physical penetration is a minimally invasive procedure that creates pores and may result in skin injury [125]. As a result, non-invasive, low-cost, and low skin-damaging transdermal administration methods should be further developed to overcome the delivery issue.

To date, little is known about the role of EVs’ cargo in skin regeneration and rejuvenation. It is critical to identify the specific cargo of EVs that is beneficial in skin improvement and ascertain its mechanism of action. Besides, most proof-of-concepts were conducted in animals, while only two human studies have been reported to date. Therefore, more clinical studies should be performed as the efficacy of EV therapeutics will likely differ between animal and human skins. However, many challenges must be overcome before proceeding to clinical application (Table 6). The areas that need further study include the scalable production of EVs, the storage and stability of EVs, therapeutic doses of EVs, the safety profile of EVs, the identification of EV cargo, and the mechanism of action of EVs. Besides, research must also address the issue of uniformity in EV production. Multiple factors can affect the quality and quantity of EVs produced by the cultured cells, including cell sources (e.g., cell types and donor selection) and culture environment (e.g., oxygen tension and temperature). Besides, the isolation methods used and the storage conditions have also been found to influence EV heterogeneity [126]. Therefore, it is critical to develop a standardized protocol of EV production and set the quality release criteria (including EV characterization and functional analysis) to attain reproducible clinical results.

Lastly, EV-based products naturally fall under biologics/drugs due to EV’s biologically active components and their ability to modify cell behavior. To remain true as a cosmeceutical product, such cell modifying effect will have to be restricted to the skin. Manufacturers will have to prove how much of these EVs get absorbed into the vascular system and the systemic effects they exert. However, cosmeceutical products are not regulated in the same manner across the globe; in fact, there has been always a gray zone between cosmetics and biologics/drugs. They are viewed as cosmetic products in Europe (EU) and Japan and regulated as drugs in the United States (US) [3]. In any case, EVs will first need to be registered as a new ingredient for cosmetic use. However, due to the broad variation of EV cargos depending on the cell source and condition that they are derived from, EV should not be used as a generic term. The responsibility lies with the manufacturer or supplier of the active ingredient to register their EVs along with their toxicokinetic and safety profile. Moreover, human-derived ingredients are prohibited from use in cosmetics in EU, US, and Malaysia due to concerns over the transmission of prions and viral diseases like human immunodeficiency virus (HIV) [127]. Additional tests and controls will be required to allay these concerns. Under the current regulation, the cosmetic industry can easily develop a cosmetic product as compared to drugs/biologics, but these may constitute a safety concern to consumers, whereas regulating a subclass of drugs/biologics provides more assurance for consumer safety and product quality but comes at a significant financial burden for the cosmetics industry. Therefore, a new and clear regulatory framework for cosmeceutical products should be developed to distinguish them from the regular cosmetics and the highly regulated drugs. This will ensure that the safety and efficacy of cosmeceutical products are monitored, thus safeguarding the benefits of consumer without causing undue hardship to the cosmeceutical industry.

## 7. Conclusions

EVs can be applied to promote anti-aging, anti-pigmentation, and scarless wound healing. They can be administered in combination with other techniques such as micro-needling and hydrogel to achieve a better result. Apart from mammalian EVs, plant EVs also demonstrated good biological activities that are suitable for cosmeceutical applications. Nonetheless, obstacles such as low yield, inconvenient storage, short shelf-life, insufficient clinical data, and the lack of understanding of the appropriate dosage, the exact EV cargoes, and their mechanism of action are hindering translation and commercialization of EV-based cosmeceuticals. As a result, improved and standardized guidelines and more research are needed to better understand the effect of EVs on skin and successfully promote the use of EVs in the facial aesthetics industry.

## Figures and Tables

**Table 1:** Evidence and clues of EVs in promoting scarless wound healing, anti-aging and skin regeneration, and anti-pigmentation.

Effect	Source of EVs	Model Used	Treatment Dose	Administration Method	Evidence and Clues	Reference
Scarless wound healing	ADSC-Exo	In vivo: Full-thickness dorsal wound in BALB/c mice model	1000 µg/mL	IV	↑ ratio of Col III:Col I, TGF-β3:TGF-β1, MMP-1 & MMP-3:TIMP-1 More flattened epidermal surface Well distributed collagen in the dermis with less cross-linking	[36]
		In vitro: Fibroblasts	0, 25, 50 & 100 µg/mL	-	↓ myofibroblast differentiation ↓ α-SMA & Col1A1 ↑ TGF-β3, Col3A1, MMP-1 & MMP-3	
	ADSC-Exo	In vivo: Excisional wound model in BALB/c mice	700 μg/mL	SC	↓ wound area ↓ Col I, Col III & α-SMA	[33]
		In vitro: HSF	20 μg/mL	-	↓ proliferation & migration ↓ Col I, Col III & α-SMA	
	ADSC-Exo	In vivo: Hypertrophic scar model in New Zealand rabbits	0.1 mL	Local injection	↓ α-SMA & Col I ↓ myofibroblast aggregation	[44]
	ADSC-Exo	In vivo: Full-thickness wound in BALB/C mice	1000 µg/mL	SC	↑ angiogenesis ↑ re-epithelialization rate ↓ scar area ↑ dermis thickness & collagen deposition	[45]
		In vitro: HDF	0, 25, 50 & 100 μg/mL (Gene and protein expression analysis & Quantification of growth factor) 0, 50, and 100 μg/mL (proliferation & migration assay)	-	↑ Col I, Col III, MMP-1, bFGF & TGF-β1 level ↓ α-SMA ↑ proliferation & migration	
	ADSC-MV	In vivo: Full-thickness wound in BALB/C mice	50 μL	SC	↑ re-epithelialization ↑ collagen deposition ↑ neovascularization ↓ wound edge (scar)	[46]
		In vitro: HUVEC, HaCaT, HDF	0, 5 & 10 μg/mL (migration assay) 0 & 20 μg/mL (proliferation, angiogenesis assay, gene and protein expression analysis)	-	↑ proliferation, migration & angiogenesis ↑ fibronectin in HUVEC, HaCaT & HDF ↑ Col I, Col III & elastin in HDF	
	UCB-MSC-Exo	In vivo: Full-thickness excisional wound in SD rats	200 μg/mL	Tail vein injection	↑ wound closure ↓ scar formation ↑ skin appendages regeneration ↑ angiogenesis Regulated collagen fibers distribution	[47]
		In vitro: HDF	25 ng/mL	-	↓ α-SMA & Col I ↑ proliferation & migration	
	UCB-Exo	In vivo: Full-thickness wound in C57BL/6 mice	2000 μg/mL	Local injection	↑ re-epithelialization ↑ angiogenesis ↓ scar width	[48]
	UC-MSC-Exo	In vitro: Fibroblasts	50, 75 & 150 μg/mL	-	↓ Col I, Col III & α-SMA	[49]
	UC-MSC-Exo	In vivo: Full-thickness excisional wound in BALB/C mice	1000 μg/mL	SC	↑ epidermal re-epithelialization & dermal angiogenesis ↓ α-SMA	[50]
		In vitro: HaCaT	125, 250, 500, 1000 ng/mL	-	↑ proliferation & migration ↓ apoptosis	
	hAEC-Exo	In vivo: Full-thickness excisional wound in SD rats	25, 50 & 100 μg/mL	SC	Well-organized collagen fibers ↑ re-epithelialization	[51]
		In vitro: HDF	25, 50 & 100 μg/mL	-	↓ Col I & Col III ↑ MMP-1 & TIMP-1 ↑ proliferation & migration	
	hAFSC-Exo	In vivo: Full- thickness excisional wound in SD rats	200 µg/mL	SC	Smoother wound edge ↑ hair follicle regeneration ↓ collagen fiber deposition ↑ nerve & vessel reconstruction ↑ cutaneous cell proliferation ↓ α-SMA, Col1A2, TGF-β1 & TIMP-1 ↑ Col3A1, TGF-β3, MMP-1 & MMP-3	[52]
		In vitro: HDF	10 & 25 ng/mL	-	↓ α-SMA	
	TSG-6 modified MSC-Exo	In vivo: Full-thickness wound model in C57BL/6J mice	1000 µg/mL	SC	↓ MCP-1, TNF-α, IL-1β, IL-6, TGF-β1, Col I, Col III & α-SMA	[32]
	iPSC-MSC-Exo	In vitro: HaCaT, HDF	10 & 20 µg/mL	-	↑ proliferation & migration ↑ Col1A1, elastin & MMP-1 in HDF ↑ Col1A1 & elastin in HaCaT	[53]
	BM-MSC-Exo	In vitro: HaCaT, HDF, EAhy926 line, Human monocytic cell U937	N/S	-	Not changes in TNF-α release by activated macrophages ↑ angiogenesis ↑ proliferation & migration of skin cells ↓ Col I, Col III, α-SMA, MMP-2 & MMP-14 as well as ↑ MMP-13 expression in myofibroblasts at the gene level ↑ Col I expression of myofibroblasts at the protein level ↑ decorin & fibronectin expression of fibroblasts at the protein level	[54]
	BM-MSC-Exo	In vivo: Full-thickness excisional wound in SD rat	250 μg	IV	↑ wound closure Restore skin function ↑ angiogenesis ↓ TGF-β1	[20]
		In vitro: HaCaT, HDF	25 μg/mL	-	↑ proliferation	
Anti-aging and promoting skin regeneration.	ADSC-Exo	In vivo: Photoaging SD rats	100 μL	SC	↓ epidermal thickness ↑ dermal thickness	[55]
		In vitro: HDF	12.5, 25, 50, 100 & 200 μg/mL	-	↑ Col I ↓ Col III, MMP-1 & MMP-3	
	ADSC-EV	In vivo: Photoaging BALB/c nude mice	150 & 300 μg/mL	SC	↓ skin wrinkle ↑ epidermal cell proliferation ↓ macrophage infiltration & ROS production	[15]
		In vitro: Photoaging HDF, 264.7 cells	50, 100, 150 & 200 μg/mL (HDF activity) 100 & 200 μg/mL (Macrophage differentiation, gene expression analysis & protein expression analysis)	-	↑ HDF activity & protected HDFs from UVB-induced senescence ↓ Col I ↑ MMP-3 ↓ M0 to M1 differentiation of macrophages ↑ SOD-1 & CAT Rescue HDFs from cell cycle arrest	
	ADSC-Exo	In vitro: UVB- irradiated HDF	N/S	-	↑ cell migration & proliferation ↓ MMP-1, -2, -3 & -9 ↑ Col I, II, III, V & elastin ↑ TIMP-1 & TGF-β1	[56]
	ADSC-Exo	In vitro: HDF	N/S	-	↓ UVB-induced DNA damage, ROS production & MMP-1 ↑ procollagen type I	[57]
	UC-MSC-EV & Fb-EV	In vitro: HDF	0, 0.5 & 5 μg/mL	-	↓ ROS production ↑ proliferation ↑ GPX-1 & Col I ↓ MMP-1 Protect cells against UVB- induced cell death & cell cycle arrest Protect cells against UVB-induced photoaging through antioxidant activity	[58]
	iPSCs-Exo	In vitro: Photoaging and naturally senescent HDF	20 × 10^8^ particles/mL	-	↓ cell damage ↓ SA-β-Gal & MMP-1/3 ↑ Col I	[59]
	TB-Exo	In vitro: Intrinsically/extrinsically senescent HNDF	1 × 10^4^ & 1 × 10^5^ particles/mL	-	↑ cell migration & proliferation ↑ Col I, Col III, elastin & fibronectin	[60]
	UC-MSC-Exo	Ex vivo: Photodamage skin model in SD rat	200, 400 & 600 µg	-	↓ skin photodamage	[61]
		In vitro: HaCaT	600 µg	-	Protect cells from oxidative stress ↓ ROS production ↑ SIRT1 expression under oxidative stress condition Activate autophagy by delivery of 14-3-3ζ protein	
Anti-pigmentation	Mouse keratinocyte-Exo	In vitro: Mouse melanocytes	N/S	-	↓ TYR, TYRP1, TYRP2 & MITF ↓ melanin content	[62]
	hAMSC- Exo	In vitro: B16F10 cells	N/S	-	↓ TYR, TYRP1, TYRP2 & MITF ↓ melanin content ↑ LC3II ↓ p62	[63]
	UVA-exposed melanocyte-EV	In vitro: Keratinocytes, melanoma cells	N/S	-	↑ BCL-xL & BCL-2 in keratinocytes ↓ PDCD4 & PTEN in keratinocytes ↑ proliferation & migration of keratinocytes and melanocytes	[64]

Abbreviations: Mesenchymal stem cell, MSC; Human amnion epithelial cell-derived exosome, hAEC-Exo; human amniotic fluid stem cells-derived exosome, hAFSC-Exo; Adipose-derived stem cell-extracellular vesicle, ADSC-EV; Adipose-derived stem cell exosome, ADSC-Exo; Adipose-derived MSC multivesicle, ADSC-MV; Bone marrow-derived MSC exosome, BM-MSC-Exo; Umbilical cord-derived MSC exosome, UC-MSC-Exo; Umbilical cord blood-derived MSC exosome, UCB-MSC-Exo; Umbilical cord blood-derived exosome, UCB-Exo; Induced pluripotent stem cell-derived MSCs-exosome, iPSC-MSC-Exo; Induced pluripotent stem cell-derived exosome, iPSC-MSC-Exo; Trophoblasts-derived exosome, TB-Exo; Fibroblast-extracellular vesicle, Fb-EV; Human amniotic mesenchymal stem cells derived exosome, hAMSC-Exo; Mouse keratinocyte derived exosome, Mouse keratinocyte-Exo; Ultraviolet A-exposed melanocyte-derived extracellular vesicle, UVA-exposed melanocyte-EV; Tumor necrosis factor stimulated gene-6 modified MSC-Exo, TSG-6 modified MSC-Exo; Sprague-Dawley, SD; Human umbilical vein endothelial cells, HUVEC; Human dermal fibroblasts, HDF; Hypertrophic scar tissue-derived fibroblasts, HSFs; Human normal dermal fibroblast, HNDF; Murine melanoma cell line from a C57BL/6J mouse, B16F10 cell; Immortalized human keratinocytes, HaCaT; Immortalized Human Vascular Endothelial Cells, EAhy926 line; monocyte-like cells, 264.7 cells; Ultraviolet, UV; ultraviolet B-rays, UVB; Intravenous, IV; Subcutaneous injection, SC; Not stated, N/S; Collagen, Col; Col1A1, Collagen type I alpha 1 chain; Col1A2, Collagen type I alpha 2 chain; Col3A1, Collagen type III alpha 1 chain; Matrix metalloproteinase, MMP; Tissue inhibitors of matrix metalloproteinase, TIMP; Transforming growth factor beta, TGF-β; Alpha-smooth muscle actin, α-SMA; basic fibroblast growth factor, bFGF; Tumor necrosis factor-alpha, TNF-α; Monocyte chemoattractant protein-1, MCP-1; Interleukin, IL; Reactive oxygen species, ROS; Superoxide dismutase type 1, SOD-1; Catalase, CAT; Glutathione peroxidase 1, GPX-1; Senescence-associated-β-galactosidase, SA-β-Gal; Sirtuin 1, SIRT1; Zeta, ζ; Microphthalmia-associated transcription factor, MITF; Tyrosinase, TYR; Tyrosinase-related protein, TYRP; Microtubule-associated protein 1A/1B-light chain 3-phosphatidylethanolamine conjugate, LC3-II; Tumor protein 62, p62; B-cell lymphoma-extra-large, BCL-xL; B-cell lymphoma 2, BCL-2, Phosphatase and tensin homolog, PTEN; Programmed cell death protein 4, PDCD4.

**Table 2:** Physical penetration methods to enhance topical penetration of EVs.

Source of EVs + Treatment	Model Used	Treatment Dose	Administration	Result	Effect	Reference
ADSCs-EVs + MN	In vivo: UV-induced photoaging model in SKH-1 mice	200 μL	Topical application	Least wrinkles Highest collagen density Organized collagen fibers ↓ cell infiltration ↓ epidermis thickness ↑ stratum corneum hydration ↓ TEWL ↑ recovery from the MN-induced injury	Anti-aging	[89]
UC-MSC-Exo + SHSs	In vivo: UV-induced photoaging model in Kunming mice	1 mg/mL	Topical application	↑ skin absorption of exosomes ↓ microwrinkle & epidermis thickness	Anti-aging	[90]
	In vivo: Guinea pigs	1 mg/mL	Topical application	Slightly irritating, but fast recovery		
3D HDF spheroids-Exo + needle-free injector	In vivo: UVB-induced photoaging model in nude mice	N/S	Needle-free injection	Least wrinkles Most dense collagen fibers Most compact stratum corneum ↓ epidermal thickness	Anti-aging	[69]

Abbreviations: Adipose-derived stem cell extracellular vesicle, ADSC-EV; Umbilical cord-derived MSC exosome, UC-MSC-Exo; Three-dimensional Human Dermal Fibroblast Spheroids-derived exosome, 3D HDF spheroids-Exo; Microneedle, MN; sponge *Haliclona* sp. spicules, SHSs; Ultraviolet, UV; ultraviolet B-rays, UVB; Not stated, N/S; trans-epidermal water loss, TEWL.

**Table 3:** Hydrogel/biomaterials-based wound dressings with EVs.

Source of EVs + Treatment	Model Used	Treatment Dose	Administration	Results	Effect	Reference
ADSC-Exo + Alg hydrogel	In vivo: Full-thickness excisional wound model in Wistar rats	300 µL	Topical application	Cumulative release of Exo from hydrogel up to 172 h ↑ wound closure, collagen synthesis & angiogenesis ↓ wound size	Promote wound healing	[94]
SR-sEVs + BSSPD hydrogel	In vivo: Full-thickness skin defect model in rats	1 × 10^11^ particles/mL	Topical application	Faster wound healing ↑ vascularization & angiogenesis Improve collagen fiber arrangement ↓ Col I/III collagen ratio Attenuated M2 polarization in later phase of wound healing	Promote scarless wound Healing	[17]
	In vivo: Rabbit ears	1 × 10^11^ particles/mL	Topical application	More ordered collagen arrangement ↓ scar elevation index		
hEnSCs-Exo + CS-glycerol based hydrogel	In vivo: Full-thickness wound model in BALB/c mice	100 µg/mL	Topical application	↑ epidermal & skin appendages formation ↑ vascularization & angiogenesis ↑ wound closure ↓ wound size	Promote wound healing	[95]
hP-MSC-EV + CS hydrogel	In vivo: Natural aging FVB mice (48 weeks old)	750 μg/mL	SC	↑ skin appendages & epithelial thickness ↑ wound closure ↑ collagen bundles ↓ SA-β-gal ↑ Col I & Col III ↓ MMP-1, 2, 3 & 9 ↑ TIMP-1 & 2	Skin rejuvenation	[68]
UC-MSC-Exo + CMCS/P407 hydrogel	In vivo: Full-thickness skin defect in SD rats	20 μg/mL	Topical application	85% cumulative release of Exo after 72 h ↑ wound closure rate, number of dermal appendages & collagen deposition, Well organized collagen fiber ↓ TNF-α & IL-1β	Promote wound healing	[96]
HUVEC-Exo + GelMA hydrogel	In vivo: Full-thickness wound model in SD rats	1 × 10^8^ particles/mL	Topical application	Controlled release of Exo until day 7 ↑ re-epithelialization, collagen alignment, deposition and maturity, & granulation tissue thickness ↑ Col I & Col III ↑ angiogenesis	Wound repair and regeneration	[97]
L929-EV + FG	In vivo: Full-thickness wound model in C57BL/6 mice	5000 μg/mL	Topical application	↑ wound closure rate ↑ collagen formation & maturation ↑ skin appendages & angiogenesis Minimum scarring	Promote scarless wound healing	[98]
EPSC-Exo + HydroMatrix	In vivo: Full-thickness skin defect in SD rats	100 μg/mL	Local injection	↑ nerve and vessel regeneration ↑ skin appendage regeneration ↓ myofiber formation ↑ Col III ↓ Col I & TGF-β1	Promote scarless wound healing	[99]
PVC-EV + HydroMatrix	In vivo: Full-thickness skin defect in SD rats	100 μg/mL	Local injection	↑ wound contraction ↓ wound size ↑ α-SMA and TGF-β1 ↑ angiogenesis & VEGF	Promote wound healing	[12]

Abbreviations: Adipose-derived stem cell-derived exosome, ADSC-Exo; Epidermal stem cell-derived exosome, EPSC-Exo; Human umbilical vein endothelial cell-derived exosome, HUVEC-Exo; Human endometrial stem cell-derived exosome, hEnSCs-Exo; Umbilical cord-derived MSC-derived exosome, UC-MSC-Exo; Sequential release small extracellular vesicle, SR-sEV; L929 murine fibroblast cell line-derived extracellular vesicle, L929-EV; Human placental mesenchymal stem cells-derived extracellular vesicle, hP-MSC-EV; Perivascular cell-derived extracellular vesicle, PVC-EV; Alginate, Alg; Bilayered thiolated alginate/PEG diacrylate, BSSPD; Carboxymethyl chitosan, CMCS; Poloxamer 407, P407; fibrin gel, FG; Gelatin methacryloyl, GelMA; Chitosan, CS; Sprague-Dawley, SD; Subcutaneous injection, SC; hour, h; Collagen, Col; Matrix metalloproteinase, MMP; Senescence-associated-β-galactosidase, SA-β-Gal; Tissue inhibitors of matrix metalloproteinase, TIMP; Transforming growth factor-beta, TGF-β; Tumor necrosis factor, TNF; Interleukin, IL; Smooth muscle alpha-actin, α-SMA; Vascular endothelial growth factor, VEGF.

**Table 4:** Potential of plant-derived EVs in cosmeceutical applications.

Source of EVs	Model Used	Treatment Dose	Administration	Result	Effect	Reference
EV from *C. fragile* and *S. fusiforme*	Clinical: 21 women (ages between 20 and 50 years)	5 µg/mL	Topical application	↑ skin brightness	Whitening	[16]
	Ex vivo: MelanoDerm tissue	5 µg/mL	-	↓ melanin synthesis		
	In vitro: MNT-1 cells	0,5, 25, 50 μg/mL (*C. fragile*) 0, 10, 50, 250 μg/mL (*S. fusiforme*)	-	↓ α-MSH-mediated melanin synthesis ↓ MITF, TYR & TYRP1		
LEV and SEV from *D. morbifera*	Ex vivo: Neoderm-ME	10 µg/mL	-	Lighter color ↓ melanin distribution in the epidermis	Anti-melanogenic	[85]
	In vitro: B16BL6 melanoma cells	1, 5 & 10 µg/mL (Melanin content measurement) 10, 50 & 100 µg/mL (TYR, TYRP-1, TYRP-2 & MITF protein or activity measurement)	-	↓ melanin content ↓ TYR, TYRP-1, TYRP-2 & MITF		
A-EV	In vitro: HaCaT & HDF	1, 5, and 10 × 10^8^ particles/mL (cell viability and SOD activity) 10^8^ and 10^9^ particles/mL (Scratch wound assay)	-	Non-cytotoxic ↑ migration ↑ Nrf2, HO-1, CAT & SOD	Antioxidant & skin regeneration	[107]
GrEV and GcEV from *P. ginseng*	In vitro: HEK, HDF & HEM	1 & 10 µg/mL (Anti-senescence effect) 0.1, 1, 5, or 10 µg/mL (Melanin level)	-	Non-cytotoxic ↓ TYRP2, TYR & RAB27 ↑ HMGB1 ↓ SA-β-Gal ↓ TP53, CDKN1A, CDKN2A, MMP-1 & IL-8 ↓ melanin	Anti-senescence & anti-melanogenic	[110]

Abbreviations: Extracellular vesicle, EV; *Codium fragile*, *C. fragile*; *Sargassum fusiforme*, *S. fusiforme*; *Dendropanax morbifera*, *D. morbifera*; *Panax ginseng*, *P. ginseng*; Aloe vera peel-derived extracellular vesicle, A-EV; Leaf-derived extracellular Vesicle, LEV; stem-derived extracellular vesicle (SEV); Extracellular Vesicle from Ginseng root, GrEV; Extracellular Vesicle from culture supernatants of Ginseng cells, GcEV; human melanoma cells, MNT-1; Mouse cell line derived from melanoma, B16BL6 melanoma cells; Human Dermal Fibroblasts, HDF; Immortalized human keratinocytes, HaCaT; Human epidermal keratinocyte, HEK; Human epidermal melanocyte, HEM; Alpha-melanocyte-stimulating hormone, α-MSH; Microphthalmia-associated transcription factor, MITF; Tyrosinase, TYR; Tyrosinase-related protein, TYRP; Nuclear factor erythroid 2-related factor 2, Nrf2; Heme oxygenase-1, HO-1, Catalase, CAT; Superoxide dismutase, SOD; Ras-related protein 27, RAB27; high mobility group box 1, HMGB1; Senescence-associated-β-galactosidase, SA-β-Gal; Tumor protein p53, TP53; Cyclin Dependent Kinase Inhibitor, CDKN; Matrix metalloprotease, MMP; Interleukin, IL.

**Table 5:** Clinical studies using EVs to improve skin appearance.

Source of EVs	Model Used	Treatment Dose	Administration	Result	Effect	Reference
ADSC-Exo	In vivo: 21 female with hyperpigmentation, aged 39–55 years	2.0 × 10^10^ particles/mL	Topical application	↓ melanin levels No cytotoxicity No adverse effect	Skin brightening effect	[115]
	In vitro: B16F10 cells	2.3 × 10^9^–3.0 × 10^11^ particles/mL	-	↓ melanin contents		
ADSC-Exo	In vivo: 18 men and 7 women with atrophic acne scars, age 19–54 years, 12 with Fitzpatrick skin type III and 13 with type IV	9.78 × 10^10^ particles/mL (for the day of FCL treatment) or 1.63 × 10^10^ particles/mL (for days subsequent to FCL treatment)	N/S	↓ ECCA scores ↓ IGA scores ↓ atrophic scar volume, mean pore volume & skin surface roughness Milder adverse effects on Exo treated side which resolved within 5 days.	Scar reduction	[116]

Abbreviations: Adipose-derived stem cell exosome, ADSC-Exo; Murine melanoma cell line from a C57BL/6J mouse, B16F10 cell; Not stated, N/S; Investigator’s Global Assessment, IGA; échelle d’évaluation clinique des cicatrices d’acné, ECCA.

**Table 6:** Challenges in translation of EV therapies.

Limitation	Recommendation
Scalable production of EVs	Application of strategies such as 3D culture using a bioreactor, physical stimulation (e.g., mechanical and electrical), chemical stimulation (e.g., drugs and small molecules), genetic manipulation to modulate the EV biogenesis and release pathway, and physiological modification (e.g., hypoxia and temperature) to up-scale the EV production
Storage and stability of EVs	Innovations in formulation or lyophilization
Dosage regime	Standardize the safe and effective dose
Safety	Perform more toxicity testing in animals (rodent and non-rodent models)
Cargo and mechanism of action	Identify the protein, nucleic acid, and lipid contents, followed by bioinformatics as well as in vitro and in vivo experiments to examine the mechanism of action
Lack of clinical data	Carried out more clinical studies
Uniformity in EV production	Development of standardized EV production, isolation, and storage protocols
